# Smelling phenomenal

**DOI:** 10.3389/fpsyg.2014.00713

**Published:** 2014-07-10

**Authors:** Benjamin D. Young

**Affiliations:** Department of Cognitive and Brain Science, Ben-Gurion University of the NegevBeer-Sheva, Israel

**Keywords:** consciousness, olfaction, awareness, qualitative-consciousness, access-consciousness, phenomenal-consciousness, olfactory imagery, anosmia

## Abstract

Qualitative-consciousness arises at the sensory level of olfactory processing and pervades our experience of smells to the extent that qualitative character is maintained whenever we are aware of undergoing an olfactory experience. Building upon the distinction between Access and Phenomenal Consciousness the paper offers a nuanced distinction between Awareness and Qualitative-consciousness that is applicable to olfaction in a manner that is conceptual precise and empirically viable. Mounting empirical research is offered substantiating the applicability of the distinction to olfaction and showing that olfactory qualitative-consciousness can occur without awareness, but any olfactory state that we are aware of being in is always qualitative. Evidence that olfactory sensory states have a qualitatively character in the absence of awareness derives from research on mate selection, the selection of social preference for social interaction and acquaintances, as well as the role of olfactory deficits in causing affective disorders. Furthermore, the conservation of secondary processing measures of olfactory valence during olfactory imagery experiments provides verification that olfactory awareness is always qualitatively conscious—all olfactory consciousness smells phenomenal.

## Introduction

Smells have a profound impact upon our daily behavior and overall quality of life even in the absence of our subjectively attending to them. There is mounting evidence that qualitative olfactory consciousness occurs in the absence of conscious awareness, however, what is even more fascinating is whenever we are aware of a smell it is qualitatively-conscious as well. Thus, it will be argued that all olfactory consciousness smells phenomenal.

The paper offers a nuanced distinction between Awareness and Qualitative-consciousness that is applicable to olfaction in a manner that is conceptual precise and empirically viable. Applying this distinct to empirical literature on olfaction shows that these kinds of consciousness do not fully dissociate for the entire modality of olfaction. Olfactory qualitative-consciousness can occur in the absence of awareness, but any olfactory state that we are aware of being in is always qualitatively conscious.

Debates regarding the nature of consciousness and its taxonomic kinds, often become conceptually murky, so it is best to initially clarify the terminological usages before entering into a discussion of the empirical evidence in support of each kind of consciousness. Pre-theoretically being aware, signifies that we can subjectively report undergoing the experience, being in the relevant state, and the content of the state. For my purposes I shall use awareness, to pick out that state in which the subject can report being in a state *S* with content *p* (or if you prefer, they are conscious of undergoing experience *E* that is of, or about, object *x*). Awareness, I shall stipulate, can be understood separately from qualitative-consciousness, such that an organism is in a qualitatively conscious state when there is something that it is like for it to undergo experience *E* which is distinguishable from undergoing experience E^*^, and moreover the subject need neither be aware of being in state *S* (i.e., undergoing *E*) nor of state *S*'s content *p*.

## Refining phenomenality

Of the many treatments of consciousness, few have been as influential in consciousness studies as access and phenomenal consciousness. Block (1995) is responsible for the claim that the concept of consciousness is not a cluster concept containing different kinds of relevantly similar concepts but a mongrel containing different kinds. The two kinds that Block is keen to distinguish are Access-consciousness and Phenomenal-consciousness (1993, 1995, 2001, 2007, 2008, 2009). However, the difference between these kinds of consciousness is defintionally opaque. Semantic and definitional clarity aside, a major difficulty with the distinction between *A-consciousness* and *P-consciousness* is that sometimes these states are differentiated and identified according to their representational content as an information processing issue (Block, 1996, 2007, 2008), while at other times *P*-*conscious* states are ostensitvely defines in light of their qualitative properties (Block, 1993, 1995).

Furthermore, the difference between these kinds of consciousness has been challenged as conceptually ambiguous (Rosenthal, 2002, 2007, 2009, 2010) and incapable of scientific investigation (Kouider et al., 2012). Moreover, a review of the literature on olfaction suggests the distinction between these kinds of conscious states might not be applicable to olfaction, because the experiential nature of *A-consciousness* and *P-consciousness* differs from the other modalities based on olfaction's unique neural architecture (Stevenson, 2009).

While the distinction on offer could be encompassed within Block's framework of *A-consciousness and P-consciousness*, further modulations and refinements of his usage of phenomenal-consciousness would be required, and as currently stated qualitative-consciousness and awareness provide greater precision and clarity in demarcation the relationship between these kinds of consciousness that is substantiated by experimental evidence from olfaction contrary to Stevenson's (2009) claim. Though it might be worried that further distinctions needlessly generate greater terminological ambiguity in an already murky subject, distinguishing between awareness and qualitative-consciousness provides clarity and nuanced evidence for the dissociation and relation between the two kinds of consciousness that Block's distinction is meant to track. As such what is offered is not meant to supplant Block's theory, but supplement it in a manner that can encompass the nature of olfactory consciousness.

Block's definition of phenomenal consciousness might be interpreted in one of two ways: as referring to states that have a qualitative character for the subject and which are conscious though not reportable or not fully accessible; or to states that have a qualitative character though the subject is in no way aware of being in the state (Rosenthal, 2002, 2007, 2009). The first interpretation corresponds to Nagel's (1974) what-it-is-likeness (WiiL) when the subject is aware of being in state *S* and *S* has a qualitative character though its content is not reportable or fully accessible. Nagel's precise usage of the phrase requires that there is a WiiL for the creature undergoing the experience. The notion of a phenomenal character of experience from a subjective point of view is inherent to the concept of WiiL. The latter interpretation of *P-consciousness* corresponds to qualitative-consciousness, since the subject is unaware of being in state *S*, yet *S* has a qualitative character of experience. This later kind arguably corresponds to that supported by Block's evidence for phenomenal consciousness from subliminal vision and extinction studies (Block, 2001, 2007, 2008, 2011).

Disambiguating these two kinds of phenomenality clarifies how the distinction of qualitative-consciousness and awareness offers greater theoretical nuance in demonstrating that qualitative olfactory states can occur in the absence of subjective awareness. The fuller conception of phenomenality as a WiiL cannot be employed in providing empirical evidence for the dissociation of these kinds of consciousness as it smuggles in awareness. Assuming WiiL would muddy the first half of my thesis that olfactory qualitative-consciousness occurs in the absence of awareness, since some manner of subjective awareness is inherent to these states, and begs the question in the second half of the thesis that olfactory awareness is always qualitative. Olfactory consciousness using the distinction between awareness and qualitative-consciousness demonstrates what the original distinction was intended to capture. Qualitative-consciousness does not smuggle in any aspect of awareness and secondary processing measures can establish the phenomenality of these states in the absence of any manner of awareness.

Methodologically employing a robust notion of awareness and contrastively the thinnest conception of phenomenal (qualitative) consciousness allows for greater conceptual clarity. Qualitative-consciousness provides the starkest way of showing that phenomenality can occur without subjective awareness. Furthermore, by stripping the subjective aspect from qualitatively consciousness states there should be no worry that some residue of subjective consciousness is smuggled in when it is shown that olfactory states that we are aware of being in are always qualitatively conscious.

## Qualitative character and secondary processing measures

It is no longer controversial that unconscious states and their content can mediate behavior and be employed in sequences of information processing, thus establishing that we can undergo cognitive states in the absence of awareness is difficult but not an insurmountable feat. The real challenge is establishing that states we are subjectively unaware of being in can have a qualitative character i.e., that there is something that it is like for the subject to undergo the experience in accordance with the aforementioned discussion of qualitative-consciousness [for further discussions of qualitative character including alternative conception see Young et al. (2014) in this research topic]. Attempts at demonstrating the qualitative character of unconscious states by inference from their similarity to conscious states often encounter the worry that the qualitative aspect present in the conscious experience is simply missing in the absence of awareness. If we cannot report the qualitative character of these states what evidence do we have of their qualitative character? One strategy for ascertaining the qualitative nature of subjectively unreportable states is Quality-Space Theory, which identifies the mental qualities in terms of their perceptual roles, such that we can ascertain the qualitative nature of these states independent of conscious awareness (Rosenthal, 1991, 1999, 2005, 2010; Clark, 1993). While this approach is applicable to olfaction (Young et al., 2014), the perceptual quality of olfactory valence permits a stronger line of evidence using secondary processing measures, which establish that the qualitative property is maintained even in the absence of awareness. Odor valence together with secondary processing measures provide an objective method of ascertaining the existence of qualitative character in the absence of awareness, thereby providing the means for demonstrating that qualitative-consciousness can occur in the absence of awareness.

The difficulty is assessing whether experiential qualities are preserved from veridical odor perception through olfactory states that we are unaware of undergoing. Since the veracity of subjective self-reports is difficult to measure and question begging in this situation, secondary-processing measures might be employed to verify that the qualitative character is conserved. Secondary processes are correlated properties or incidental effects (Cummins et al., 2001), such as speed, error rate, types of errors, or fatigue etc., of the system when it performs a task. In addition to a state's performance of a role, other secondary properties can be used to evaluate whether the role was performed utilizing the same physical realization.

Secondary processing measures are traditionally employed in debates regarding computational implementations of cognitive abilities, however, analogous measures are available in measuring perceptual states. In olfactory research the property of valence (the perceive pleasant or unpleasant property of an odor) provides just such needed measures for assessing a states qualitative properties independent of awareness. Behavioral measures such as sniff rate and volume, response time, and heart rate can all be used as independent measures of perceived valence that indicate the olfactory system is treating these stimuli in the same fashion regardless of whether we can subjectively report our perception of the qualitative character. Sniff rates relative to odor concentration and valence substantiate the inference that subjectively unaware olfactory states have a qualitative character.

There is a long history of considering the primary qualities of odors to be their pleasantness or unpleasantness. Plato is the most well-known instance of the claim that smells are primarily individuated in terms of their olfactory valence (*Timaeus* 66d–67b; Plato, 1997), which is echoed within Indian Philosophy (McHugh, 2012, Ch. 2). More recently it has been argued that valence is the primitive property that determinates odor identity (Yeshurun and Sobel, 2010). Unlike the identification and categorization of odor quality that is similar but varies across cultures there is greater agreement on the categorization and identification of odors using the properties of pleasant or unpleasantness (Haddad et al., 2010). However, recent evidence suggests that though valence might be a primitive property of odor's, the object of olfactory experience is more likely identified by humans in terms of its olfactory quality and not valence (Olofsson et al., 2012). Whether olfactory quality or valence is the property that determines odor identity, valence is considered to be one of the most basic perceptible qualities possessed by odors.

Sniff rates relative to odor concentration and valence provide confirmation of an olfactory state's qualitative character. Humans modulate their sniff rate and volume 150 ms after the onset of a stimulus relative to its concentration and valence (Johnson et al., 2003). The stimulus dependent response of human sniffing is such that intense and unpleasant odorants are sniffed less vigorously and with a decreased volume. Measurement of olfactory motor responses to odorants is reliable enough to be used as a non-verbal measure of human's detection and categorization of the odor (Frank et al., 2003). Additionally, anosmics show no such response indicating that the sniff response only occurs in accordance with the subject's experiencing the valence of the presented stimulus (Harland and Frank, 1997). Sniff rate and volume are not the only secondary measures for assessing odor valence. Response time is faster in detection and discrimination tasks for unpleasant odors (Bensafi et al., 2003a) and heart rate measurements show that we involuntarily categorize unpleasant odors (Bensafi et al., 2002).

In what follows it will be noted whenever secondary processing measures of odor valence can be used to establish that the state has a qualitative character. Furthermore, it will be argued that this aspect of olfactory processing together with the distinction of qualitative-consciousness and awareness allows a nuanced treatment of consciousness that can empirically support the claim that we can undergo qualitative experiences in the absence of awareness.

## Olfactory sensory states are phenomenally conscious

Olfactory sensory states have a qualitative character even in the absence of awareness. Evidence that olfactory sensory states have a qualitatively character in the absence of awareness, derives from research on mate selection, the selection of social preference for social interaction and acquaintances, as well as the role of olfactory deficits in causing affective disorders^1^. While none of these phenomena are decisive on their own and further research is certainly required, when taken together they provide a host of initial evidence indicating that qualitative-consciousness can arise independently of awareness.

### Mate selection

Evidence for the qualitative character of olfactory sensory states can be gleaned from research on mate selection. Further research on human olfactory mate selection is required, but the initial data indicates that mate selection in humans is influenced by smell (reviewed in Havlicek and Roberts, 2009)^2^. We might not be aware of it, but our reason for choosing sexual partners might be that their immune system smells pleasant to us.

Using olfactory cues we select mates based on the synergy of our combined immune systems for producing stronger offspring. If we mate with a partner whose major histocompatibility complex (MHC, alternatively termed human leukocyte antigen, HLA, in humans) is the converse of our own, this generates offspring with a more robust hybrid immune system. Thus, it is adaptive to be able to detect the structure of a possible mate's MHC.

However, the difficulty of studying human mate selection is readily apparent given our inability to control for intervening variables. Most studies examining HLA mate choice have proven inconclusive, which could be attributed to these studies being conducted in heterogeneous populations in which the confounding effects of ethnic or racial self-preference could not be controlled. Nonetheless the importance of smell in mate selection cannot be discounted. Based on questionares rating the factors of mate selection, female subjects rated body odor as one of the most important factors in selecting sexual partners (Herz and Cahill, 1997; Herz and Inzlicht, 2002).

The qualitative character of a prospective mate's body odor plays a role in determining our choice of sexual partners, but to establish that this is related to odors derived by our HLA compounds, as detected by the olfactory system, and these mediate actual mate selection requires three steps. First, it will be shown that humans can detect and discriminate the same MHC compounds that determine olfactory mate selection in rodents. Second, it will be shown that we have the ability to detect the olfactory signature of HLA compounds and that these are treated as having a qualitative property. Lastly, the literature of actual human mate selection in relation to HLA compatibility will be selectively reviewed.

The causal mechanism for HLA detection is arguably the same as the mechanism responsible for MHC detection and recognition in animal models. Odors derived from MHC compounds play a role in determining mate selection in rodents. In mice and rats it has been demonstrated that MHC recognition is accomplished by the olfactory system (Yamazaki et al., 1979, 1980; Ehman and Scott, 2001). Further research has also shown that mice, rats, and humans can smell the difference between the urinary scents of rodents derived from different MHC strains of mice (Beauchamp et al., 1985). Taken together, these studies show that mammals certainly employ MHC-based mate selection and that the human olfactory system is sensitive to these same chemicals. When these findings regarding our olfactory sensitivity are combined with the research on human mate selection, strong evidence emerges that we engage in HLA-based mate selection as mediated by olfactory cues, in the same manner as other mammals.

Odors derived from our HLA not only mediate mate selection, but it can be shown that these odors have a qualitative character. Using two-day-old sweaty t-shirts of men, experimenters determined that females judge a t-shirt's odor most pleasant when it was derived from a man whose HLA system differed from their own (Wedekind et al., 1995; Jacob et al., 2002). In both these studies no single male body odor was universally agreed to be pleasant smelling; hedonic judgments differed across females relative to the dissimilarity of the donor's HLA. The major difference between these studies is that in Wedekind et al.'s study the more dissimilar the HLA, the stronger the hedonic rating; while Jacob's results displayed a degree of HLA overlap in paternal lineage implicated in the hedonic rating of the sweaty odor. Nevertheless, both studies clearly implicate the olfactory system as a possible means for selecting mates based on the qualitative character of body odor as determined by HLA.

However, these positive results at best establish a correlation effect between the MHC of the donor and judged pleasantness. Recently the work of Aksenov et al. (2012) demonstrated that MHC yields volatile odor compounds (VOC) at the cellular level. Their study was the first to demonstrate that MHC compounds give off unique detectable odor signatures, such that a change in a single allele produces unique odor fingerprint at the cellular level. The implication of these results is that each person unique genetic makeup and in particular HLA complex will generate VOCs with a unique odor signature, thus allowing the connection between the judged hedonic profile of complimentary HLA mates and the possibility that this is directly determined by the VOC generate by a person's MHC compounds.

Further evidence that humans can detect the odor profile generated by the HLA complex can be found in studies of perfume selection. Pre-theoretic intuitions suggest that humans perfume themselves to mask their body odor, since body odor on its own is commonly perceived as unpleasant. However, Milinski and Wedekind (2001) disproved the masking hypothesis by showing that we select perfumes that enhance our natural body odor. Not only is this effect only found for the self-selection of fragrances, which is explained by the fact that people usually purchase fragrances for themselves (Jellinek, 1951; Le Norcy, 1991), but also that the judged pleasantness of an odor as correlated with body odor was consistent over a 2 year period and not a matter of changing fashion.

In addition to a perfumes enhancement of the pleasantness of perceived body odor, Lenochova et al. (2012) discovered that a self-selected perfume boosted the judged pleasantness of body odor relative to each person, as shown by their control that presented a mixture of body odor and equally pleasant perfume that had not been selected by the subject did not generate the same judged odor enhancement. However, it should be noted that their study was only conducted with male subjects. Since, female body odor is generally less intense making it more likely prone to a masking effect, further research was required.

Recently Milinski et al. (2013) used female subjects to address this concern and replicated their previous findings that we can select perfumes based on the MHC profile of oneself but not others. Fragrances similar to the VOC given off by ones own MHC have a boosting effect on body odor. Moreover, using fMRI imaging Milinsky et al.'s study revealed specific activation to peptides consistent with humans' ability to detect MHC associated olfactory cues. Thus, HLA compounds generate VOC with a qualitative character that we can detect and behaviorally respond to.

The strongest evidence that human mate selection preferences are driven by avoidance of those with HLA haplotypes identical to ours is derived from Oberet al.'s (1997) study of Hutterite mate choice. Previous studies did not show an effect of HLA on mate selection, but were conducted in heterogeneous populations where olfactory factors of mate selection might have been overridden by socio-economic and ethnic factors. The Hutterite population served as a control, because it is a small homogenous population with easily traceable genetic lineages. By looking at the HLA haplotype matches between spouses, they concluded that less of an overlap existed than would otherwise be expected if the selection processes were random. Ober et al. concluded that MHC based mate choice is operant even in humans. Furthermore, they suggest that the mechanism for HLA detection and structural comparison might be mediated by the olfactory system. The olfactory system is quite capable of such chemical structural analysis and comparison, as demonstrated by the aforementioned results that humans can detect and discriminate the relevant MHC odorants in rodents, are sensitive to MHC compounds of their own body odor, and judge body odors of complimentary HLAs as more pleasant.

The Ober et al study is by far the most significant source of data on the role of MHC in actual mate choice in humans, because of its methodological soundness using a large sample within a closed homogenous population thereby controlling for social and ethnic confounds. Of the studies on the role of MHC in mate choice only four have shown that MHC is significant in determining actual mate choice (Giphart and D'Amaro, 1983; Rosenberg et al., 1983; Ober et al., 1997; Chaix et al., 2008), while seven have shown no significance (Pollack et al., 1982; Nordlander et al., 1983; Sans et al., 1994; Jin et al., 1995; Ihara et al., 2000; Garver-Apgar et al., 2006). However, it should be noted that aside from the most recent study (Chaix et al., 2008) which showed a limited effect in only their European American grouping, the previous studies with positive results all used large sample sizes, thus controlling for the variegated properties of genetic variation as well as additional societal and normative practices in selecting mates. The null results of previous studies might simply be attributed to lack of power due to small sample sizes in attempting to determine a complex human behavior with multiple intervening variables.

Most recently Chaix et al. (2008) showed that MHC mate selection is apparent in European and American populations, but not in African Yoruba populations. However, the statistical methods of testing their hypothesis was criticized, because the significance could be attribute to extreme mate pairs within the groups, as well as for not correctly adjusting their statistical thresholds for multiple hypothesis testing (Derti et al., 2010). After adjusting their previous results for multiple hypotheses (Laurent and Chaix, 2012), the critics agreed (Derti and Roth, 2013) that MHC based mate selection was an apparent, but not a robust result, which might simply be attributed to the small sample size. Further research is certainly called for on the role of VOC given off by MHC compounds in humans in the selection of mates across cultures. Currently the evidence indicates that odorant detection of MHC compounds influences sexual mates selection, but the extent and mechanism require further study using more stringent and universal methodologies with large samples (Havlicek and Roberts, 2009).

The argument put forward in this section is that VOCs derived from HLA have properties with qualitative character that are perceived using the olfactory system and modulate our mate selection behavior. Yet, we are not commonly aware of smells in general (Sela and Sobel, 2010) nor their specifically modulation our selection of mates. Further research is required using the secondary measures of odor valence (i.e., sniff rate and volume, response time, and heart rate) relative to the subliminal presentation of olfactory stimuli derived from the VOC of similar and dissimilar sets of HLA subjects to fully establish that HLA mate selection occurs in the absence of awareness based on genuinely qualitative states. However, at this initial stage the evidence strongly suggests that we select mates based upon the qualitative character of our olfactory states even in the absence of awareness.

### Social acquaintance selection

Further evidence that olfactory sensory states have a qualitative character in the absence of awareness, can be derived from research on olfaction's effect in guiding social preferences. While it is uncontroversial that our awareness of perceived smells modulate our mood and affective responses toward people (Herz and Schooler, 2002; Jacob et al., 2002), subliminally pleasant and noxious odors can modulate our ratings of the likeability of social acquaintances (Li et al., 2007). Li et al.'s study showed that the valence of an odorant subliminally modulates social preference. Using a simple odor detection task (pleasant, unpleasant, neutral, and control) combined with a subjective rating of the likeability of pictures of faces, they demonstrated that pleasant and unpleasant odors presented subliminally, both had a physiological effect and modulated the subject's affective response toward pictures of human faces.

Independent of subjective awareness there was a significant change in the heart rate of each subject relative to the valence of the subliminal odors, thereby confirming the qualitative character of these states employing secondary processing measures. Furthermore, unpleasant odorants caused the subject to rate the face as being less likable, while pleasant odorants had the opposite effect. The modulation of likability relative to odorant valence only occurred with subliminal odorants and quickly disappeared if the subject was aware of the smell. Even in the absence of subjective awareness the odorant has a qualitative property of valence, which has a causal effect upon our predication of qualitative properties to others. Arguably this shows that qualitative-consciousness is independent of our subjective awareness of the pleasant or unpleasant character of the odor. Even if one is unaware of undergoing an olfactory experience, the valence of subliminal odorants are implicated in social acquaintance selection. I might like you, because you smell nice.

### Anosmia—argument from absence

A severely unethical, but clearly conclusive, experiment could be performed to test whether qualitative-consciousness arises at the sensory level of olfactory processing in the absence of awareness. The experiment would be to sever the olfactory tract in healthy humans to see if they could undergo qualitatively-conscious olfactory states. Though this experiment is ethically unfeasible some olfactory pathologies provide subjects with similar deficits that suggest it is not possible to have olfactory qualitative-consciousness without olfactory sensory states.

Anosmia is the most common disorder of olfactory pathology in which individuals lose their sense of smell. In some cases anosmia is due to the presence of a psychological disorder, but the vast majority of cases result from damage to the olfactory bulb either due to infection or head trauma. Individuals with fully functional olfactory systems modulate their sniffing in accordance with the pleasant or unpleasant character of an odor, yet anosmics show no such response (Harland and Frank, 1997) demonstrating that the sniff response only occurs when the subject perceives the valence of the presented stimulus. Thus, using secondary processing measures it is arguably the case that anosmic individuals lack the ability to perceptually experience the qualitative character of olfactory valence.

In addition to their inability to perceive olfactory stimuli, anosmic individuals also experience a decrease in their hedonic quality of life (Miwa et al., 2001) and motivational anhedonia (Keller and Malaspina, 2013) that is often causally implicated in the further development of depression (Deems et al., 1991)^3^. We are not aware of our olfactory experiences most of the time, but they imbue our lives with a qualitative character of experience, which is most striking in their absence.

To summarize, the Argument from Absence is that the absence of olfactory sensory states and anosmics inability to experience the qualitative valence of odors are causally implicated in lower quality of life scores and depression. Hence, these states are responsible for generating qualitative-consciousness even in the absence of awareness. The argument might not prove that all olfactory sensory states have a qualitative character, but the evidence certainly is significant and nicely fits with the mounting evidence thus far.

## No olfactory awareness without qualitative-consciousness

Evidence for the claim that olfactory awareness is always qualitatively-conscious might be derived from first-person reports and the reader's own awareness of olfactory experiences. Introspecting, remembering, or imagining an odor, tokens some manner of qualitative olfactory experience. Just thinking about the smell of the fresh cut grass elicits an olfactory experience for me. However, using first-person reports of phenomenology might be methodologically questionable. Aside from biasing us to only consider experiences that we are aware of as having a qualitative character, the veracity of olfactory first-person reports might be doubted given our limited attention to olfactory experience and subsequent lack of awareness of our experience of odors (Sela and Sobel, 2010).

Veridical odor perception could establish that anytime we are aware of an olfactory experience it has a qualitative character, but it is not a good test case. Situations of perceiving olfactory stimuli will activate a sensory state, which the previous section argued are qualitatively conscious, thereby making one aware of an olfactory quality. Consequently, anytime we are aware of perceiving an odor, the conscious state has a qualitative character, because qualitative sensory states are elicited as part of creating the perceptual state. Because first-person phenomenological reports are methodologically questionable and perceptual states might always have a qualitative character, olfactory imagery will serve as the test case for the conditional claim that if we are aware of an olfactory state then it must be qualitatively-conscious as well.

Methodologically one could exhaustively search for a case in which we are perceptually aware of an odor and yet the experience does not have any qualitative character. However, a stronger and more fatal test of my claim would be to find a state, such as olfactory imagery that is not perceptual, that we commonly do not think would be qualitative, and that people find difficult eliciting in the first place (Herz, 2000) and check if these cases of olfactory awareness are qualitatively conscious. High-level cognitive states concerning olfactory experience are paradigmatic test cases of conscious awareness where we would not expect some level of qualitative character. What will be shown is that just thinking about odors, even those that we have not previously experienced, will elicit a qualitative character of experience.

While the phenomenon of olfactory imagery is primarily conceived as an issue regarding the representational format of cognitive states in an analogous manner to visual imagery (Kosslyn, 2003; Kosslyn et al., 2003; Pylyshyn, 2003), it demonstrates that we can elicit a qualitative experience of a smell in the absence of an olfactory stimulus (reviewed in Rinck et al., 2009). Olfactory imagery demonstrates that all states of olfactory awareness are also qualitatively-conscious. Experimentally it has been shown that subjects can elicit the qualitative experience of smelling something in the absence of olfactory stimuli. Merely introspecting, imagining, or thinking about a smell elicits a qualitative experience of smelling an odor.

Even more fascinating is that olfactory imagery states mimic those of ordinary olfactory experiences such as odor mixing (Algom and Cain, 1991). Odor mixing experiments yield the interesting results that, when two similar odorants are combined to yield a configural compound, the resulting complex's odor is different from those of its constituents parts, while odorants that are dissimilar yield elemental compounds in which the odors of the constituents are clearly discernable. However, by simply changing the concentrations of the constituents, one can shift an elemental compound to a configural compound. What is of interest in olfactory imagery is that if one is asked to imagine the mixture of two odors and report the olfactory quality of the compound, the reports will mimic those given when smelling the actual odor.

However, for olfactory imagery to fully demonstrate that states of olfactory awareness are qualitative-consciousness it must be shown that these state's content and experiential properties are the same as the perceptual state. The most obvious way to test for such an overlap of content and qualities would be based on self-reports as employed in Algom and Cain's (1991) study, yet these must be marginalized for the same reasons as introspective reports of past olfactory experiences—we simply cannot methodically test the veracity of subjective self-reports regarding olfactory imagery (Djordjevic et al., 2004).

Self-reports are doubtless invaluable tools, but they must be corroborated with other measures of the content and qualitative character. If olfactory imagery is to demonstrate that whenever we are aware of an olfactory experience there is a qualitative character of experience what needs to be shown is that these imaginary creations of an olfactory state have the same experiential properties as if the subject where perceiving the imagined stimulus. A review of the literature on olfactory imagery suggests that this can be demonstrated using the similarity of sniff patterns between veridical perception and olfactory imagination. The sniffing patterns are similar between both types of experiences suggesting that to elicit an olfactory qualitative experience one must manipulate the olfactory epithelium and bulb (the sensory states), which then recreates the experience by activating the olfactory cortex (Djordjevic et al., 2005; Bensafi et al., 2007; Rinck et al., 2009). To think about a smell, one must token the initial sensory and perceptual states, which are arguably qualitatively conscious.

However, a more recent set of experiments (Tomiczek and Stevenson, 2009) calls this into question and argues that the same perceptual state is not elicited, rather similar structures that are utilized for olfactory perception in general are activated. Tomiczek and Stevenson (2009) assert that we do not imagine a specific odor, rather there is a general overall increase in activation across areas in the olfactory system that are responsive to odorants similar to the imagined odor. While their results indicate that the imagine state does not have the same exact content it does focus us in the right direction. Though these states might not be fully identical, secondary processing measures can be utilized to establish that the best explanation of their content must involve qualitative character.

The methodology of verifying the qualitative character of an imaginary mental experience as being the same as veridical perception using measures of sniffing is currently employed in olfactory imagery studies. Using olfactory motor activity during imagery as a criterion to test the veracity of participants claimed imagined olfactory percept, Bensafi et al. (2003a,b) confirmed that the same sniff parameters including sniff volume occur in imagery as in conscious veridical perception. They not only showed that sniffing is sensory dependent, but also sniffing in a similar fashion to veridical perception produce qualitatively more robust olfactory imagery (Bensafi et al., 2005; Bensafi and Rouby, 2007). Employing the same secondary processes increased the capacity for generating olfactory images and the strength of the olfactory quality indicating that these subjects had olfactory experiences with qualitative character.

Kleemann et al. (2009) lend further support to the conservation of sniff rates as indicating the preservation of the same olfactory quality of experience and extended them to breathing patterns. The overall sniff volume and breathing amplitudes are the same between imaginary and perceptual olfactory states. Subjects not only reported an ability to imagine an odor in these experiments, they also breathe and sniff in the same fashion as if they actually perceived the odor. Moreover preventing subjects from sniffing while imagining smells decreases the vividness of the imagined smell (Arshamian et al., 2008). These results further solidify the claim that olfactory imagery states are contentful cognitive states with qualitative character.

Additional secondary measures of response time and heart rate lend further confirmation of the qualitative nature of these imagined states. Response time is faster in detection and discrimination tasks for unpleasant odors (Bensafi et al., 2003a) and heart rate measurements show that we involuntarily categorize unpleasant odors (Bensafi et al., 2002). Given the role of sniffing in modulating olfactory imagery it is unsurprising that olfactory imagery increases our detection rate of the target odor in a manner that is modality and content specific (Djordjevic et al., 2004, 2005). The subject's experience of odor valence during olfactory imagery can be verified using behavioral non-verbal measure such as sniff patterns and response time. These secondary measures establish the occurrence of the qualitative experience of valence in olfactory imagery.

The preservation of secondary measures of sniff-rates (as well as other behavioral measures) enables the further inference that the experiential quality is being conserved in olfactory imagery. However, even with the corroborations of secondary measures it might still be objected that the subjects are merely employing their tacit knowledge of olfactory perception in generating their reports and behavior during these experiments.

Similar criticisms have been used against visual imagery, however critiques of this variety gain no traction in the case of olfactory imagery. The sniff responses in these cases make it absurd to claim that these states might be merely modulate by our propositional knowledge of olfactory perception, but contain no actual qualitative character. It seems fanciful that we could modulate our breathing and sniffing patterns in such a precise and automatic manner when we barely pay attention to these facets of our olfactory experience in normal cases of perceptions. Furthermore, our olfactory experiences are arguably not formatted in the same fashion as our descriptive linguistic resources (Young et al., 2014). If olfactory perceptual experiences and memories that we are conscious of are not formatted in the objections prescribed descriptive format it would be rather surprising if the same format was not preserved in olfactory imagery. Moreover, it has been shown that an increase in overall Anhendonia decreases our ability for olfactory imagery (Bensafi and Rouby, 2007; Rouby et al., 2009) thereby implicating some level of qualitative character in mediating olfactory imagery.

We can cognitively generate an olfactory experience that has an olfactory quality mimicking veridical perception in terms of its subjective report, behavioral measures, physiological responses, and cortical activation (Bensafi et al., 2007; Rinck et al., 2009). The fact that these states conserve and preserve all of these properties from veridical perception indicates that olfactory imagery states have a robust olfactory qualitative character. Thereby supporting the claim that any time there is olfactory conscious awareness these states are also qualitatively conscious.

## Conclusion

Even while we are unaware of it, a world of odors continually envelops us exerting a profound influence on our behavior and the qualitative character of our everyday experiences. These smells contribute to the quality of our life and have a qualitative character such that it is possible for one to be in a qualitative olfactory state, but not be aware that one is undergoing the experience. What is even more controversial is that it is not possible for one to be aware of an olfactory experience without it having a qualitative character.

Olfactory qualitative-consciousness occurs in the absence of awareness, as demonstrated by research on social acquaintance selection, mate selection, and the Argument from Absence derived from the anosmic's decreased quality of life measures. Thus, the occurrence of olfactory qualitative-consciousness in the absence of awareness is compatible with Block's treatment of phenomenal consciousness and shows his distinction to be applicable to olfaction. The second line of evidence that all states of olfactory awareness are qualitatively-conscious suggests that the dissociation between these kinds of consciousness differs from expectations derived from vision studies. Further research is certainly called for, but at this initial stage of inquiry it seems plausible that qualitative-consciousness plays a constitutive role in the formation of olfactory awareness as these states arise at the sensory level and are elicited whenever we either have an awareness of an occurent odor experience or attempt to recollect, imagine, or think about olfactory experiences.

### Conflict of interest statement

The author declares that the research was conducted in the absence of any commercial or financial relationships that could be construed as a potential conflict of interest.

## Footnotes

^1^Blind smell is an olfactory phenomenon reminiscent of aspects of Blind Sight that is not nearly as well studied, but preliminary studies (Schwartz et al., 1994; Schwartz, 2000; Sobel et al., 1999) suggest that some healthy human subjects can detect the presence of an odor in the absence of subjective awareness. A subgroup of subjects in Schwartz et al.'s experiment provides suggestive evidence that olfactory qualitative states can occur in the absence of awareness. However, further research needs to be conducted to see the prevalence of sensitive subjects in the overall population using more robust measures for determining odor detection threshold than those employed by Schwartz et al. to ascertain that the subject was unaware of the odor stimulus. Additionally, Sobel et al.'s results are suggestive of there being dose dependent unconscious olfactory processing, but the qualitative status of these states is dubious, since their detection task employed a subliminal odor without any further measure of subjective feedback. Without further measures of the subject's experience of these stimuli the claim that these unconscious experience contained properties that are qualitative is unwarranted. This is not to discount the findings of both sets of experiments, but only to point out that further research is require before the phenomenon can provide support for the claim that olfactory qualitative-consciousness can occur in the absence of awareness.

^2^The nature and debate regarding human pheromones is irrelevant to all claims regarding the olfactory mediation of human mate selection within this paper, since all the evidence derives from olfactory perception utilizing the olfactory epithelium through higher levels of olfactory processing. The phenomena under discussion in this section does not conform to the definitional nature of pheromones and is not mediate by the veromenasal system as is the case in other mammals (for a more in depth treatment of pheromones consult Doty, 2010).

^3^These studies of anosmia do not specify the nature of the anatomical damage, since their focus is upon the resultant olfactory deficit. Thus, to fully test the claim that qualitative-consciousness occurs at the sensory level in olfaction in the absence of awareness further research needs to be conducted on anosmia resulting from a severed olfactory tract.
